# Photobiomodulation as an antioxidant substitute in post-thawing trauma of human stem cells from the apical papilla

**DOI:** 10.1038/s41598-021-96841-3

**Published:** 2021-08-30

**Authors:** Woori Choi, Ku Youn Baik, Seung Jeong, Sangbae Park, Jae Eun Kim, Hong Bae Kim, Jong Hoon Chung

**Affiliations:** 1 Department of Biosystems Engineering, Seoul National University, Seoul, 08826 Republic of Korea; 2 Electrical and Biological Physics, Kwangwoon University, Seoul, 01897 Republic of Korea; 3 Department of Biosystems & Biomaterials Science and Engineering, Seoul National University, Seoul, 08826 Republic of Korea; 4 Research Institute for Agriculture and Life Sciences, Seoul National University, Seoul, 08826 Republic of Korea

**Keywords:** Biochemistry, Biological techniques, Biophysics, Biotechnology, Cell biology, Physiology, Stem cells

## Abstract

Cryopreservation, the most common method of preserving stem cells, requires post-processing because it produces trauma to the cells. Post-thawing trauma typically induces cell death, elevates reactive oxygen species (ROS) concentration, and lowers mitochondrial membrane potential (MMP). Although this trauma has been solved using antioxidants, we attempted to use photobiomodulation (PBM) instead of chemical treatment. We used a 950-nm near-infrared LED to create a PBM device and chose a pulsed-wave mode of 30 Hz and a 30% duty cycle. Near-infrared radiation (NIR) at 950 nm was effective in reducing cell death caused by hydrogen peroxide induced-oxidative stress. Cryodamage also leads to apoptosis of cells, which can be avoided by irradiation at 950 nm NIR. Irradiation as post-processing for cryopreservation had an antioxidant effect that reduced both cellular and mitochondrial ROS. It also increased mitochondrial mass and activated mitochondrial activity, resulting in increased MMP, ATP generation, and increased cytochrome c oxidase activity. In addition, NIR increased alkaline phosphatase (ALP) activity, a biomarker of differentiation. As a result, we identified that 950 nm NIR PBM solves cryodamage in human stem cells from the apical papilla, indicating its potential as an alternative to antioxidants for treatment of post-thawing trauma, and further estimated its mechanism.

## Introduction

As the use of stem cells in cell therapeutics and tissue engineering is increasing rapidly, preservation of stem cells has become an important issue. Most stem cells are stored by cryopreservation using cryoprotective agents to preserve the fine structure of cells during freezing and thawing. These processes can produce changes in physical and chemical properties^1^. Despite development of such agents, cryopreservation still causes post-thawing trauma to cells^2^, including a lower proliferation rate^3,4^, decreased mitochondrial membrane potential (MMP)^5^, decreased differentiation^6,7^, greater deoxyribonucleic acid (DNA) damage^8^, increased reactive oxygen species (ROS)^6^, and decreased motility in sperm^9^. One of the main causes of these problems is oxidative stress^10^, which collapses the cellular redox system and leads to apoptosis^11^. Antioxidants that reduce oxidative stress caused by post-thawing trauma have emerged as a solution^2^. In general, antioxidants significantly reduce DNA oxidation and lipid peroxidation in cryodamaged cells. However, not all antioxidants resolve post-thawing trauma^2^. Antifreeze proteins did not affect mitochondrial activity in mouse oocyte^12^, and the antioxidant coenzyme Q10 was not effective in restoring MMP^13^. Antioxidants had no effect on reduced ATP production caused by post-thawing trauma in murine germ cells^14^.

Photobiomodulation (PBM) is an application of certain wavelengths of light to biological systems to change their cellular activity. Applied PBM involves a wide spectrum from 600 to 1100 nm to reduce inflammation, edema, and pain and regenerate damaged tissues^15^. In these reactions, cytochrome c oxidase (CCO) acts as the primary cellular photoacceptor. According to a previous study, PBM often induces a dose-dependent increase in ROS production in biological systems but decreased ROS levels in cells exposed to oxidative stress at 750 and 950 nm^16^. Therefore, PBM can be used as a substitute for antioxidants in post-thawing trauma to overcome the limitations of chemical antioxidants.

PBM is divided into two modalities: continuous wave (CW) and pulsed wave (PW). CW involves continuous irradiation of light, while PW involves square pulsed wave irradiation. Production of ROS varies between CW and PW^17,18^. At the same energy, ROS generation is much higher in PW than CW^19^. Moreover, generation of ROS depends on the frequency and duty cycle of PW^20^. Responses to PW differ by target cell type. To identify the optimal pulsed wave, delayed luminescence (DL) is needed. DL is a measure of the intensity of light emitted from cells after the light source is turned off. The emitted photons are a response to modulation of ROS production by PBM. Thus, DL represents mitochondrial activity in the cells^19^. To determine the optimal effectiveness of PBM on modulation of ROS production, it is necessary to determine the decay time of DL to resolve post-thawing trauma.

In this study, we used human stem cells from the apical papilla (SCAP). DL was used to determine the optimal PBM condition for all following measurements. In such conditions, ROS was measured to verify that 950 nm Near-infrared radiation (NIR) PBM effectively resolved cryodamage induced-oxidative stress. Apoptosis was measured to determine if oxidative stress induced by cryodamage increases apoptosis and if reduction of oxidative stress by 950 nm NIR PBM decreases apoptosis. Since there are various causes of post-thawing trauma, the hydrogen peroxide-treated cells were irradiated by 950 nm NIR, and cell viabilities were measured. The thawed cells were dyed with Mito-SOX and MitoTracker to determine how 950 nm NIR PBM affects the concentration of mitochondrial ROS. CCO assays were performed to show if CCO activity was activated or inhibited, and ATP was measured to verify change in ATP generation. rtPCR was implemented to identify the mRNA expression affected by ATP (which acts as a transcription factor) produced by 950 nm NIR PBM. Alkaline phosphatase (ALP) activity was measured with an ALP assay after osteogenic induction to determine if 950 nm NIR PBM has an additive effect in thawed cells. We collated these results to establish a hypothesized mechanism for 950 nm NIR PBM.

Recently, PBM was applied in frozen sperms and showed better cell activity^21–23^. However, mechanistic studies of PBM repairing post-thawing trauma are insufficient. Antioxidants usually used to reduce oxidative stress during thawing cells showed limitations in recovering mitochondrial activity. Since the predominant PBM mechanism is the activation of CCO as a photoreceptor in mitochondria, we hypothesized that PBM on thawing stem cells could restore mitochondrial function and differentiation potential^24^. We confirmed that PBM has a positive effect on cryodamaged stem cells and established the hypothesis that increased oxygen consumption of CCO increases mitochondrial activity. The results of this study will improve the efficiency of stem cell preservation by reducing the proportion of stem cells damaged by cryopreservation.

## Results

### Optimization of PBM conditions

**Figure 1:**
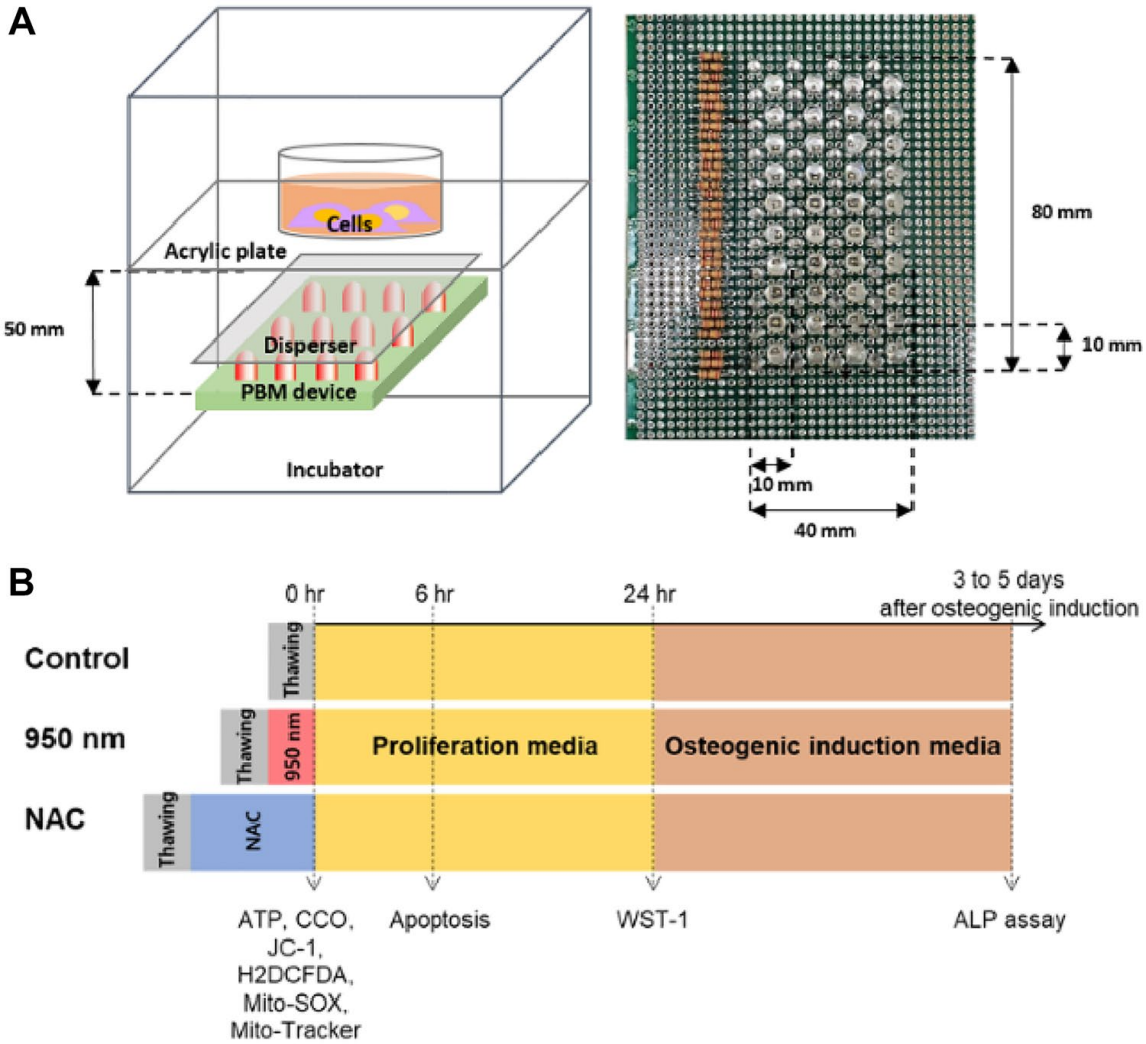
Schematics of the PBM device and experimental time schedule. (**A**) The PBM device for this study. The light source is placed in a 37 °C incubator to block light other than the light source. The 950 nm LEDs are placed at 10 mm intervals, and a disperser is used to produce uniform irradiation. The transparent acrylic cell culture plate is placed 50 mm above the LEDs. (**B**) Timelines of the experiments. All frozen cells were thawed in a 37 °C water bath for 2 min. All measurements, except those of apoptosis, were performed immediately after cells were irradiated with 950 nm NIR or treated with N-acetylcysteine (NAC). Apoptosis of cells was measured after 6 h.

**Figure 2:**
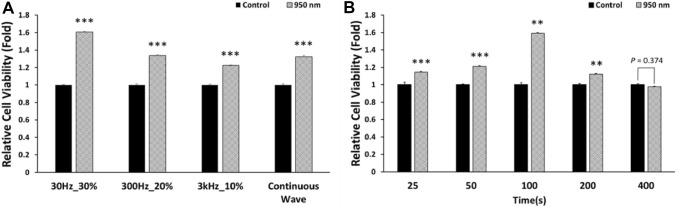
Optimization of 950 nm NIR irradiation conditions for cell viability. (**A**) Relative cell viability at energy density of 175.68 mJ/cm^2^. Four irradiation conditions were chosen to test a variety of frequencies, duty cycles, and decay times of DL. (**B**) Relative cell viability with different energy densities. Light with the same frequency and duty cycle, 30 Hz and 30%, was applied with different exposure times. The energy density of the 950 nm NIR irradiation for 100 s was 175.68 mJ/cm^2^. Data are mean ± standard deviation (SD) (n = 10). **P < 0.01, ***P < 0.001 versus controls. *P* values were determined with one-way ANOVA.

A PBM device was placed in an incubator (Fig. 1A). The timeline for all experiments is described in Fig. 1B. For DL measurements, 16 pairs of frequency and duty cycle were used. The frequency values were 30, 300, and 3000 Hz. Both PW and CW modes were used. Duty cycles of 10, 20, 30, 40, and 80% were used in PW mode. The DL showed the longest decay time for a 30 Hz frequency and 30% duty cycle and the shortest decay time for 3 kHz and 10% duty cycle (Table 1). Cell viabilities were observed for various duty cycles and frequencies of irradiation to determine the correlation of viability of irradiated cells after thawing with decay time. For viability measurements, the pairs of frequency and duty cycle with the shortest decay time, the longest time, and a moderate value were used with CW mode at the same energy density of 175.68 mJ/cm^2^ (Fig. 2A). Viability showed a significant (*P* < 0.001) increase at 30 Hz and 30% duty cycle compared with the control, a 1.6-fold increase. In contrast, viability was 1.2 times higher at 3 kHz and 10% duty cycle than in the control group. Therefore, a frequency of 30 Hz and a duty cycle of 30% were chosen for the subsequent experiments using 950 nm NIR irradiation. In the chosen conditions, cell viability was analyzed at different energy densities to determine the optimal value. Energy density is controlled by irradiation time, and 25, 50, 100, 200, and 400 s were used as experimental conditions. Accordingly, 100 s was the optimal stimulation time, with 5.856 (± 0.534) mW/cm^2^ (Fig. 2B). Cell viability was 1.6 times higher in the 100 s group than the control, and the difference was statistically significant (*P* = 0.01). Accordingly, 100 s were used for irradiation in all following experiments.

**Table 1:** Decay times by frequency and duty cycle.

	Initial intensity (a.u.)	Beta	Tau	Decay time (s)
30Hz_10%	0.8491	0.934	0.6787	1.30128197
30Hz_20%	0.7577	0.7431	0.3922	1.11421181
30Hz_30%	0.6555	0.1712	0.706	242.274563
30Hz_40%	0.0318	0.2769	0.0462	1.66404491
30Hz_80%	0.0971	0.8235	0.6948	1.64530977
300Hz_10%	0.7952	0.1869	0.4898	102.713069
300Hz_20%	0.6551	0.1626	0.119	55.6617995
300Hz_30%	0.4984	0.9597	0.3404	0.62458609
300Hz_40%	0.5853	0.2238	0.7513	64.766706
300Hz_80%	0.2551	0.506	0.6991	4.34552389
3kHz_10%	0.8909	0.9593	0.5472	1.00470911
3kHz_20%	0.1386	0.1493	0.2575	208.503088
3kHz_30%	0.8407	0.2543	0.8143	40.737337
3kHz_40%	0.2435	0.9293	0.35	0.67660439
3kHz_80%	0.1966	0.2511	0.616	32.4322556
Continuous wave	0.4387	0.3816	0.7655	9.75492925

### NIR-reduced apoptosis and ROS concentration

**Figure 3:**
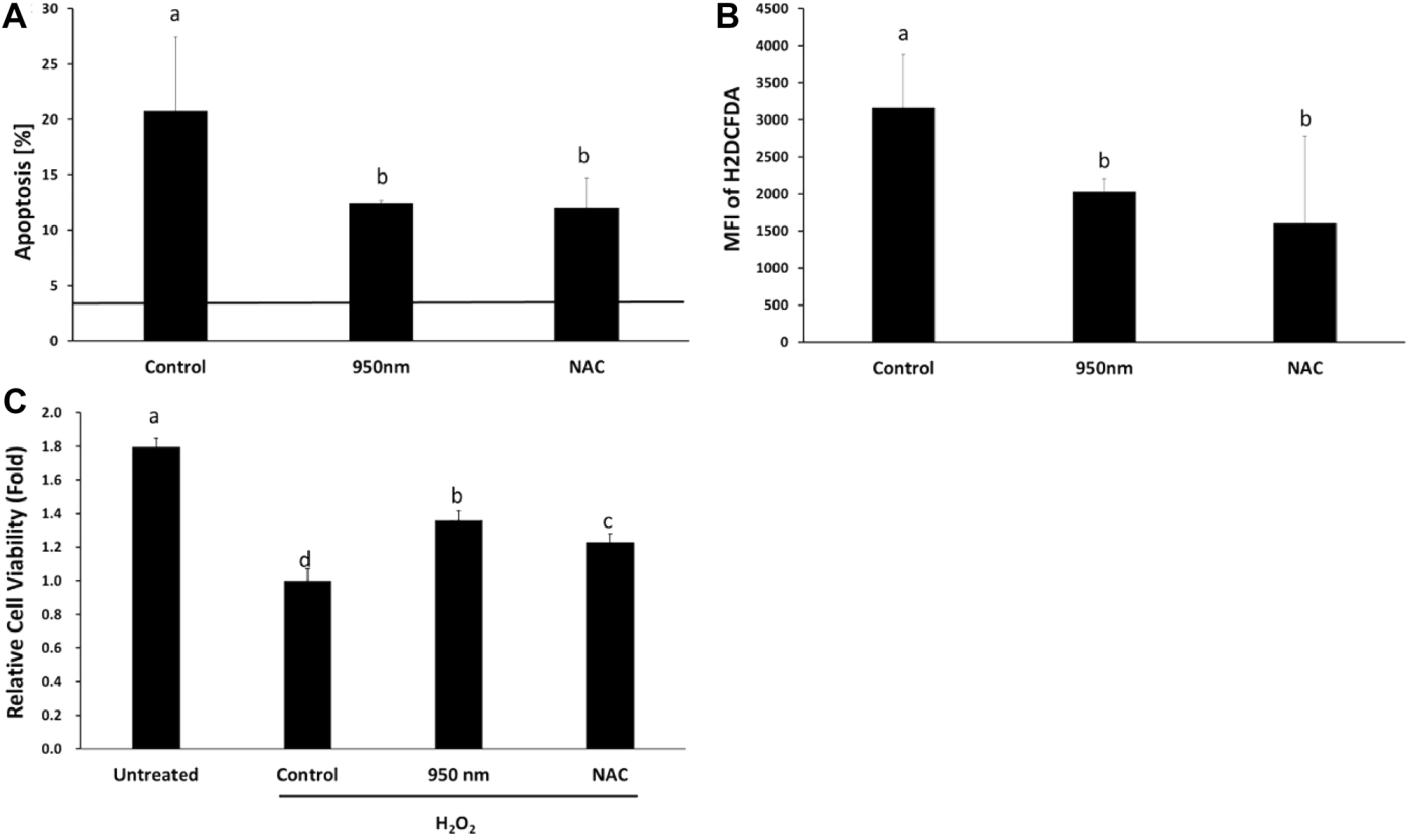
Irradiation with 950 nm NIR reduced apoptosis and ROS as NAC. (**A**) Apoptotic cell rate. The solid line on the graph is the results of the non-cryopreserved group. (**B**) MFI of H2DCFDA. (**C**) Relative viability of cells with hydrogen peroxide. The reduction of viability was alleviated by 950 nm NIR and NAC treatment. Data are mean ± SD (n > 5). Different letters at the tops of columns are significantly different at *P* < 0.05 according to Duncan’s test.

Cryopreservation induces programmed cell death^10^. Since 950 nm NIR PBM was effective in reducing cell death caused by oxidative stress, we studied whether it is effective in preventing apoptosis induced by cryodamage. An increase was observed in the ratio of cells with apoptosis by FACS. The percentage of apoptosis was 20.785% in the control, 12.447% in the 950 nm group, 12.013% in the N-acetylcysteine (NAC) group, and 3.553% in the non-cryopreserved group (Fig. 3A). Columns with different letters indicate significant (*P* < 0.01) differences according to Duncan’s multiple range test. There was a definite difference between the control and the non-cryopreserved groups, indicating that cryopreservation significantly increased programmed cell death. The irradiation of 950 nm NIR with 30 Hz and 30% duty cycle reduced cryopreservation-induced apoptosis significantly (*P* < 0.01). NAC-treatment also produced a significant decrease in apoptosis.

Frozen cells undergo explosive oxidative stress, which is one of the major reasons for cryodamage^2,25^. Median fluorescence intensity (MFI) of H2DCFDA was analyzed in each group to ensure that 950 nm NIR PBM reduces ROS concentration in thawed cells. The NAC and 950 nm group had significantly (*P* < 0.05) lower MFI than the control (Fig. 3B). The MFI of H2DCFDA and the percentage of apoptosis were proportional. Thus, increased ROS by cryopreservation was one of the main factors inducing apoptosis in post-thawing trauma. PBM with 950 nm NIR reduced both programmed cell death and ROS concentration.

### NIR-reduced oxidative stress induced by hydrogen peroxide

There are other causes of cell death in cryodamage in addition to oxidative stress. The effects of 950 nm NIR PBM on oxidative stress alone must be determined to verify that irradiation with 950 nm NIR affected cells damaged by oxidative stress. The cells were treated with 0.25 mM hydrogen peroxide to increase ROS concentration and cause oxidative stress. For comparison with normal cells, there was an untreated group. The control group did not undergo any post-processing, the 950 nm group was irradiated with 950 nm NIR, and the NAC group was treated with NAC after hydrogen peroxide treatment. The viability of SCAP was measured after 24 h of irradiation. Comparing the control group with the untreated group, the oxidative stress of hydrogen peroxide reduced cell viability by 0.56 times. The 950 nm group showed 1.36 times higher viability than the control group (Fig. 3C). The difference was statistically significant (P < 0.01). The NAC group showed significantly higher viability than the control (P < 0.01). However, the NAC group showed decreased cell viability compared to the 950 nm group. Irradiation with NIR increased the viability of cells treated with hydrogen peroxide. Thus, irradiation at 950 nm could resolve oxidative stress in place of antioxidants such as NAC.

### NIR-reduced mitochondrial ROS concentration

**Figure 4:**
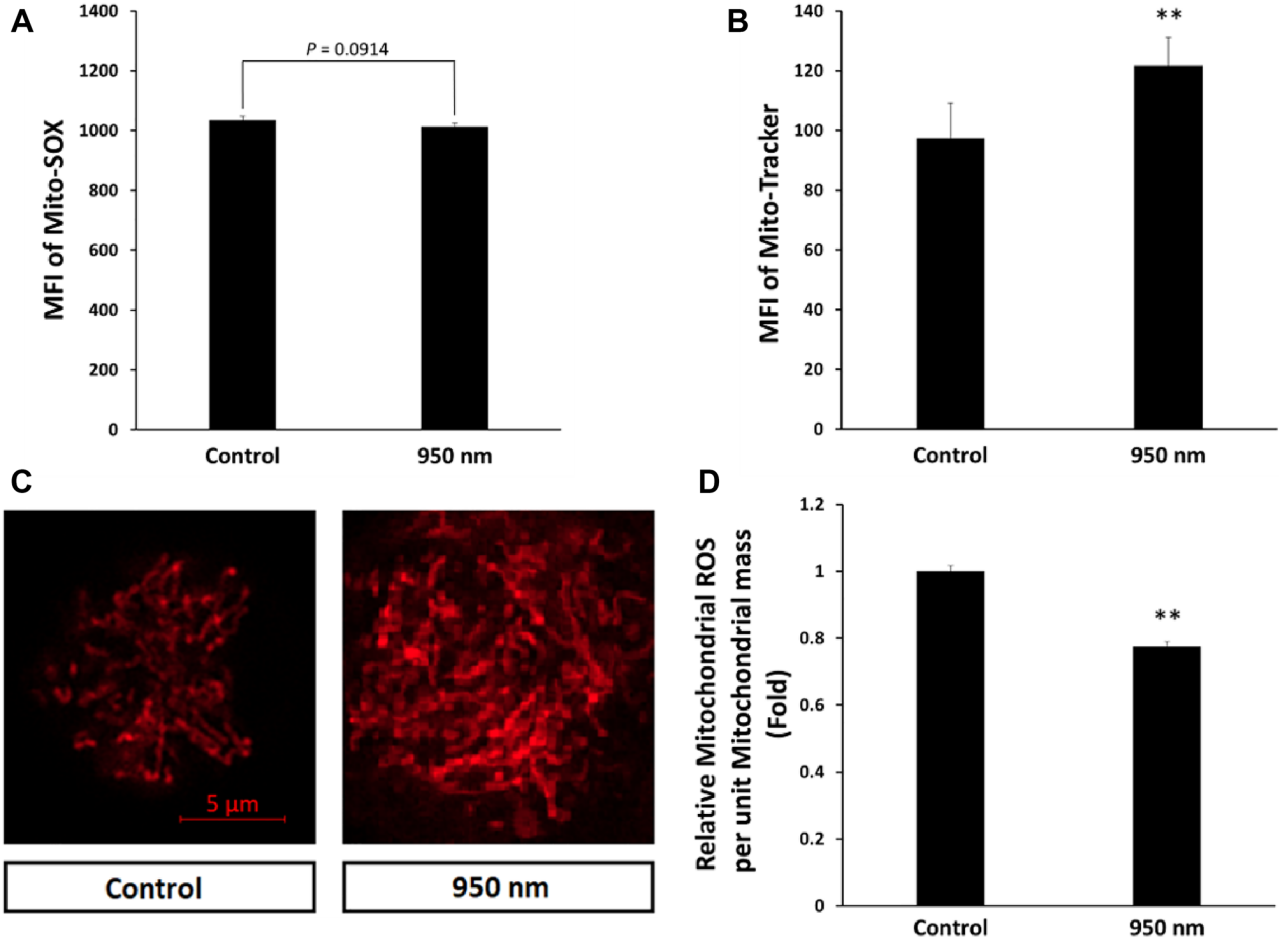
Decreased mitochondrial ROS by 950 nm NIR irradiation. (**A**) MFI of Mito-SOX (mitochondrial ROS) was slightly reduced. The two groups did not show a significant difference. Data are mean ± SD (n > 5). *P* = 0.0914 compared to control. (**B**) MFI of Mito-Tracker (mitochondrial mass) was significantly enhanced. (**C**) Typical confocal microscopic images of Mito-Tracker-stained cells. (**D**) Relative amount of mitochondrial ROS per unit mitochondrial mass. To compare the ROS in each treated group with that of the control group, the MFI of Mito-SOX was divided by that of the Mito-Tracker. The two groups showed a significant difference. Data are mean ± SD (n > 5). **P < 0.01 compared to controls. *P* values were determined by one-way ANOVA (**A–D**).

Mito-SOX (which detects only mitochondrial ROS) was used to distinguish mitochondrial ROS from cytosolic ROS. The irradiated group did not show a statistically significant difference compared with controls (Fig. 4A). However, the two groups showed a statistically significant (*P* < 0.01) difference in mitochondrial mass. Mitochondria were dyed with Mito-tracker deep red, and its MFI was measured by FACS. Mitochondrial mass is represented by the MFI of the Mito-tracker and increased 1.25 times in the 950 nm group compared to the control (Fig. 4B). A difference was seen from the results observed with a confocal microscope after Mito-tracker dying (Fig. 4C). In the 950 nm group, dyed mitochondria occupied a larger area than the control. The MFI of Mito-SOX was divided by the MFI of the Mito-tracker, and the result indicates the amount of mitochondrial ROS per unit mitochondrial mass. In the 950 nm group, the amount of mitochondrial ROS per unit mitochondrial mass significantly (*P* < 0.01) decreased by 0.77 times compared to the control (Fig. 4D). Accordingly, irradiation of 950 nm NIR resulted in ROS in cytosol and a simultaneous decrease in mitochondria.

### Effects of NIR on mRNA expressions

**Figure 5:**
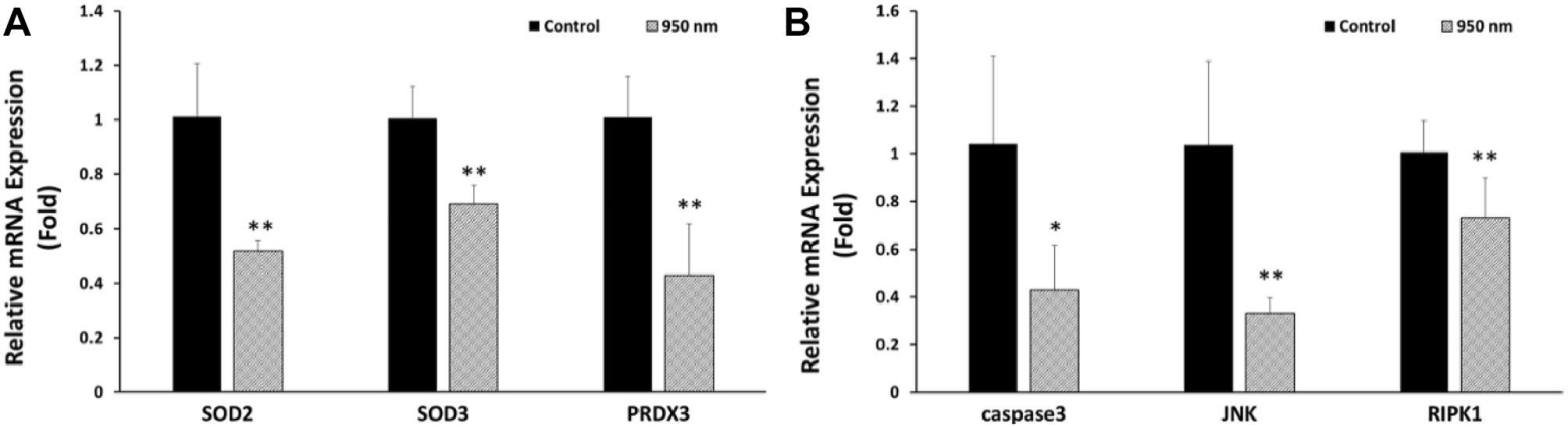
Relative mRNA expression of control and 950 nm -irradiated group. (**A**) SOD2, SOD3, and PRDX3 are related to oxidative stress. (**B**) Caspase 3, DUSP1, JNK, and RIPK1 are related to apoptosis. Data are mean ± SD (n > 5). *P < 0.05, **P < 0.01 versus controls. *P* values were determined by one-way ANOVA (**A,B**).

rtPCR was implemented to identify which mRNA expression changed under the influence of 950 nm NIR PBM. ROS- and apoptosis-related mRNAs were expressed in rtPCR. In terms of expression of ROS-related mRNAs, 950 nm NIR PBM decreased the expression of SOD2 encoding the mitochondrial ROS scavenger protein, PRDX3 encoding the antioxidant enzyme localized in the mitochondria, and SOD3 encoding the cytosolic ROS scavenger protein in thawed cells (Fig. 5A). In terms of apoptotic expression, the 950 nm group showed reduced expression of three mRNAs compared to the control group: caspase3 encoding caspase protein, JNK1 encoding JNK (which plays a role in the apoptosis pathway), and RIPK1 encoding RIP kinases. Cells irradiated after thawing showed lower expression of mRNAs related to cell death than did the control (Fig. 5B). Therefore, 950 nm NIR irradiation tends to reduce expression of mRNA related to apoptosis.

### NIR-enhanced mitochondrial activities

**Figure 6:**
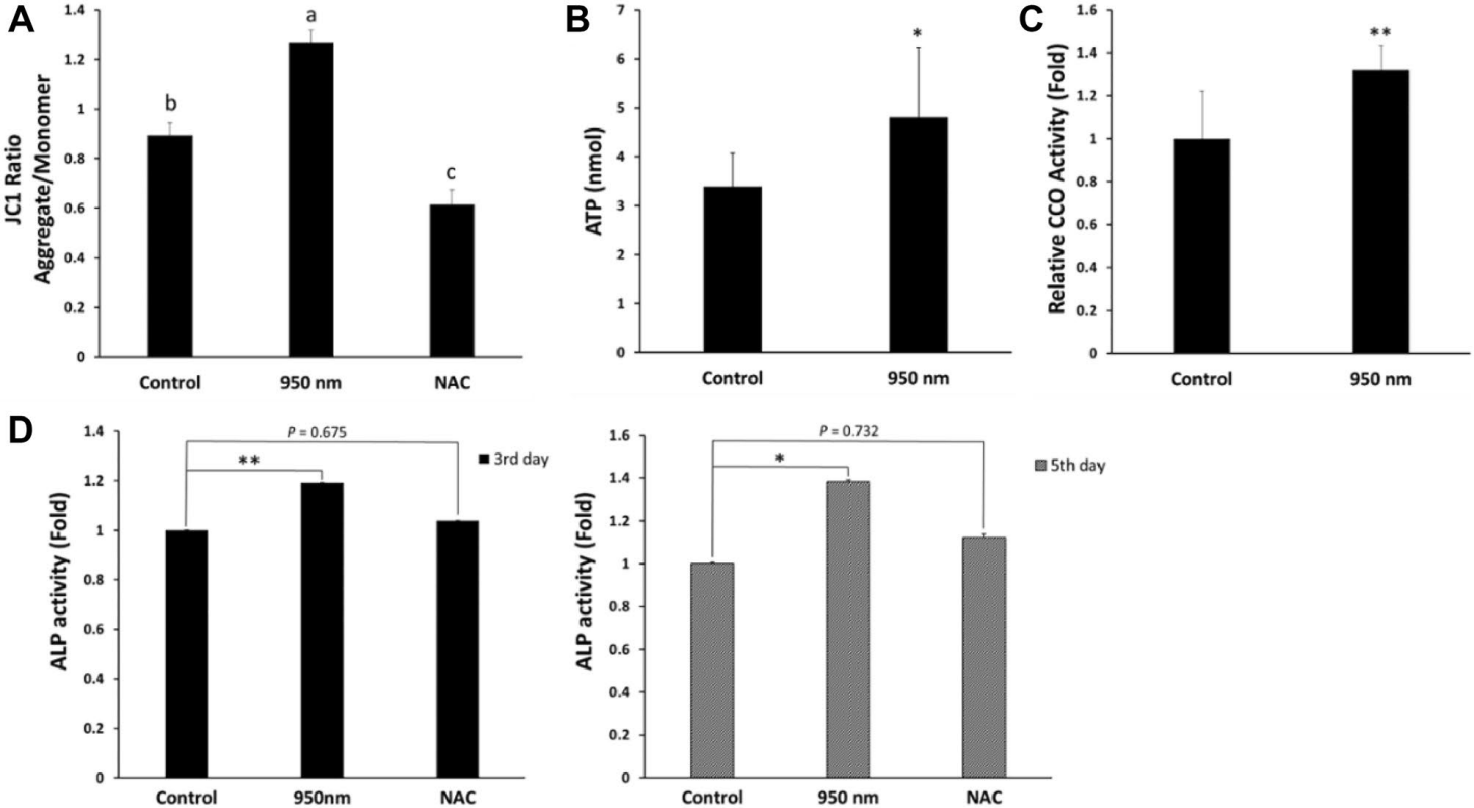
Mitochondrial activity and additive effect. (**A**) Mitochondrial membrane potential measured by JC1. The ratio of aggregate and monomer of JC1 is called the JC1 ratio proportional MMP. Data are mean ± SD (n > 5). According to Duncan’s test, different letters at tops of the columns indicate significant differences at *P* < 0.05. **(B**) Comparing the amount of ATP with the control. ATP in each group was purified from 10^6^ cells using the ATP assay kit. Data are mean ± SD (n = 10). *P < 0.05 compared to controls. (**C**) Oxygen consumption of cytochrome c oxidase. Activity of CCO for each group was measured by the CCO assay kit. Activated CCO indicates activated ETC. Data are mean ± SD (n > 5). *P < 0.05 compared to controls. (**D**) Relative ALP activity on and days 3 and 5. Data of each group are expressed as a multiple of those of the control group for the corresponding date. To verify that NIR irradiation has an additive effect of osteogenic differentiation in stem cells, ALP assays were performed on and days 3 and 5 after osteogenic induction. Data are mean ± SD (n = 10). *P < 0.05 compared to controls. *P* values were determined by one-way ANOVA (**A–D**).

Irradiation with NIR highly relates to mitochondrial activity because photons of NIR activate CCO to form complex IV of ETC in mitochondria. Moreover, 900 nm NIR stabilizes ion bonds between iron and the center of the pyrrole ring in protoporphyrin IV contained in cytochrome C^26^. MMP, ATP generation, and CCO activity were measured to analyze mitochondrial activities. First, the MMP using the JC-1 assay kit was represented by the JC-1 ratio, which is proportional to MMP^27^. This was measured in the 950 nm group, the NAC group, and the control group. Compared to the control group, the JC-1 ratio was 1.42 times higher in the 950 nm group and 0.69 times lower in the NAC group (Fig. 6A). The difference among the three groups was statistically significant (*P* < 0.05). Reduction of JC-1 ratio can represent either mitochondrial depolarization or cell death^28^. According to the procedure given in the kit, the JC-1 ratio should be analyzed with other cytotoxicity data. A comparison with apoptosis data verified that NIR prevents cell death in thawed cells by increasing MMP. Second, the amount of ATP was quantitatively obtained using the ATP assay kit in analysis of ATP generation. A significant (*P* < 0.05) 1.42-fold increase in ATP production was observed in the 950 nm group compared with the control group (Fig. 6B). Hyperpolarization of MMP and increasing ATP generation occurred simultaneously. Third, the 950 nm group and the control group were compared to determine the activity of CCO (which represents oxygen consumption). The CCO activity was 1.32 times higher in the 950 nm group than the control (Fig. 6C). The difference between the two groups was statistically significant (*P* < 0.01). Post-thawing trauma induced-inactivated CCO was activated by irradiation with 950 nm NIR.

**Figure 7:**
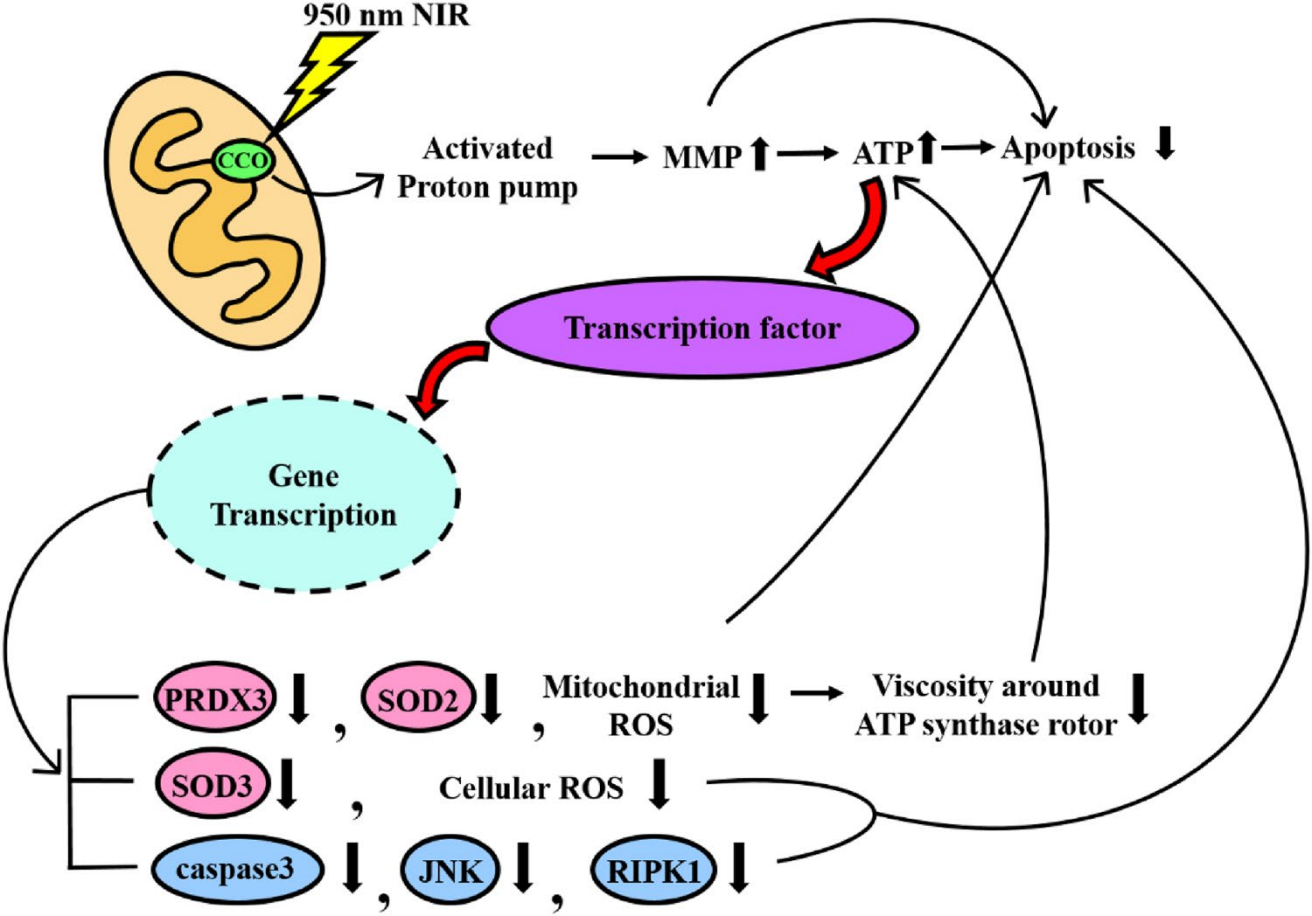
Schematic diagram of the mechanism of PBM in solving post-thawing trauma. The green color inside the mitochondria is a CCO that is activated during 950 nm NIR irradiation. The cyan circle is a nucleus in which ATP acts as a transcription factor to regulate mRNA expression. Red is ROS scavenger-related mRNA and blue is apoptosis-related mRNA.

### NIR-activated ALP activity

ALP activity was measured to verify that 950 nm NIR PBM is beneficial for differentiation in thawed stem cells. In the ALP assay, the irradiation group showed significantly (*P* < 0.01 on day 3 and *P* < 0.05 on day 5) higher ALP activity than the control (Fig. 6D). The ALP activity of the 950 nm group was 1.2 times higher than that of the control on day 3 after osteogenic induction and 1.4 times higher than the control on day 5. The NAC group did not show significant difference from the control on day 3 or 5. The 950 nm NIR irradiation was effective in increasing ALP activity, indicating differentiation (the ultimate goal of stem cells) in thawed cells.

## Discussion

Authors should discuss the results and how they can be interpreted from the perspective of previous studies and of the working hypotheses. The findings and their implications should be discussed in the broadest context possible. Future research directions may also be highlighted. The aim of this article was to verify that 950 nm NIR PBM can be used as a substitute for antioxidants in cryopreserved stem cells. Also, a hypothesis about the mechanisms of post-thawing trauma resolution by PBM was developed based on experimental results. PBM is used as a treatment for various diseases, and its effectiveness and virtues are drawing attention. It is commonly used for wound healing, recovery of cardiac ischemic injuries, and preventing deterioration of injured ophthalmic nerves^29^. Moreover, brain disorders such as traumatic events, degenerative diseases, and psychiatric disorders were treated by PBM^30^. In addition, PBM with red and NIR wavelengths served as curing skins for wound healing, pain relief, and function restoration^31^. PBM has the advantages of being cost-effective and simple to use compared to chemical treatments, which require continuous purchase of consumable drugs. Drugs require relatively complex processing and comparatively strict storage methods. PBM is a simple process of irradiation and is not difficult store. In treatments using PBM, the therapeutic effect is clearly visible, while the mechanisms have not yet been clarified. The results of this study, that PBM was effective in reducing ROS concentration, coincide well with those found in earlier experimental studies^16,32,33^. Our conclusion that applying PBM effectively improves cell activity that could be degraded post-thawing trauma is in close agreement with previous studies^21,22^. Specifically, 950 nm NIR PBM improved CCO activity in this study. This finding is not in agreement with the results of Sanderson et al.^16^ because the cell conditions used in the CCO assay were different. Unlike our study, they used normal rather than damaged cells to measure the oxygen consumption of CCO. Their other in vitro assays used OGD- and glutamate-exposed cells. This study demonstrated results and mechanisms for use of PBMs in thawed stem cells, unlike previous works. The application of PBMs to address post-thawing trauma is limited to a few studies, and most of these focused on sperm. Furthermore, the previous studies did not have clear considerations of ROS and contemplation of mechanisms.

Irradiation with a frequency of 30 Hz and a duty cycle of 30% was chosen by prescreening using DL. A longer decay time and higher viability of thawed cells were observed at a power density of 1.7568 mW/cm^2^, which is 30% of 5.856 mW/cm^2^ (Fig. 2A). This showed that 950 nm NIR was more effective in ensuring the viability of thawed cells when DL was emitted slowly with the same energy density. In hydrogen peroxide-treated cells, 950 nm NIR PBM significantly increased the viability of cells (Fig. 3C) by preventing oxidative stress-induced cell death, one of the major reasons for post-thawing trauma. This implies that PBM could be used as an alternative to antioxidants.

When thawing, the water layer temperature is lower than normal, which increases the viscosity^34^. Higher viscosity during ATP synthesis decreases ATP generation^35,36^. Thus, the viscosity around the ATP synthase can increase due to the low temperature of the thawing cell, resulting in a reduced ATP generation rate relative to normal cells. Moreover, post-thawing trauma increases ROS generation, since ROS is hydrophilic, increasing the viscosity around ATP synthase. There is a rational argument that a significant decrease in ATP level could result in cell death^37^. Measurement of ROS concentration confirmed that cryopreservation produces oxidative stress, and 950 nm NIR reduces the ROS concentration of thawed cells (Figs. 3B, 4D). There are two regions of ROS concentration measurement (cytosol and mitochondria), both of which showed reduced ROS concentration by irradiation. The reduction in mitochondrial ROS due to 950 nm NIR irradiation could lead to a decrease in viscosity and contribute to ATP generation. The increased ATP generation in the 950 nm group was measured through an ATP assay (Fig. 6B). Irradiation with 950 nm NIR increased the ATP level, which had rapidly decreased due to thawing, and reduced apoptosis (Fig. 3A). Thus, 950 nm NIR prevented apoptosis by reducing oxidative stress and relieving ATP shortage through ROS reduction.

There is also an increase in MMP (in addition to ATP increasing) after 950 nm NIR PBM, which might be a mechanism that prevents apoptosis in post-thawing trauma. A high ROS concentration depolarizes MMP^38^. Oxidative stress induced by cryodamage affects MMP. Cryopreservation increases ROS concentration and opens mitochondrial permeability transition pores (mPTPs). Opening of mPTPs leads to dissipation of MMP due to proton leakage in cryodamaged cells^2^. In some cases, the increase in ROS is accompanied by an increase in MMP. If mitochondria experience pathological problems related to ETC channel failure, MMPs decrease while ROS increase^39,40^. It is assumed that thawed cells have a dysfunctional ETC channel, resulting in MMP reduction with increase in ROS generation. Treatment with 950 nm NIR PBM hyperpolarizes MMP, which is depolarized by the opening of mPTPs and oxidative stress, maintaining homeostasis. Preventing depolarization of MMP both reduces cell death and increases ATP generation.

The increase in MMP by 950 nm NIR PBM was achieved by activating ETC. NIR activates CCO and increases oxygen consumption of CCO. It also stabilizes the bonds between iron ions and the center of the porphyrin contained in cytochrome C^26^. These activate electronic transmission in mitochondria, and proton pumps are activated by proton-coupled electron transfer^41,42^. MMP is increased due to the activated proton pump and promotes ATP generation. Sanderson et al*.* showed that 950 nm NIR inhibits CCO, which reduces ROS concentration^16,43–46^. This is contrary to the findings of our study that 950 nm NIR PBM activates CCO. The reason for the discrepancy was the cellular redox state difference in the cells before irradiation. In their study, CCO activity was extracted from cells with normal ROS levels. In our work, CCO activity was measured in cells immediately after thawing and in cells irradiated after thawing.

As previously mentioned, 950 nm NIR PBM results in an increase in ATP generation in various ways. Increased ATP acts as a transcription factor and affects the expression of some mRNAs. Expression of mRNAs associated with ROS scavenging was reduced: SOD3 of the cytosolic ROS scavenger related mRNA, SOD2 of the mitochondrial ROS scavenger related mRNA, and PRDX3 of the antioxidant enzyme related mRNA (Fig. 5A). This was consistent with the flow cytometry analysis in which both cellular and mitochondrial ROS concentrations decreased. Also, mRNA expression of caspase3, JNK1, and RIPK1 related to apoptosis signaling were decreased (Fig. 5B). Collectively, these caused a decrease in cell death.

In addition to relief of cryodamage by 950 nm NIR PBM through alleviation of oxidative stress, other mechanisms contribute. First, the mitochondrial membrane potential was increased by 950 nm NIR PBM, which restores the ATP level decreased by thawing. Also, 950 nm NIR PBM activates CCO to form ETC in mitochondria and increases the stability of bonds between iron ions and the center of the pyrrole ring in protoporphyrin IV contained in cytochrome C^26^. Stabilization of these bonds helps cytochrome C retain electrons in ETC, and the increased oxygen consumption of CCO activates ETC (Fig. 6C). Both of these increase MMP and help the cells produce ATP, preventing apoptosis induced by ATP shortage. Furthermore, 950 nm NIR PBM activated mitochondrial activity, as described previously, and increased mitochondrial mass. It is likely that the 950 nm group had more mitochondria than the control because of the small number of mitochondria destroyed by mPTP opening due to the cryodamage. PBM using 950 nm NIR is an advantageous strategy for processing oxidative stress after thawing in oocyte cryopreservation, where mitochondrial activity and preservation of mitochondria are particularly important. In addition, proper mitochondrial function is critical to differentiation of stem cells^47,48^. Thus, 950 nm NIR irradiation after thawing was effective in improving ALP activity through mitochondrial function normalization. Decreased mitochondrial mass and excessive mitochondrial ROS hinder mitochondrial function^49^. However, 950 nm NIR PBM increased mitochondrial mass, which is decreased during thawing, and reduced mitochondrial ROS. Mitochondrial dysfunction inhibits osteogenesis, increases osteoclast activity, and accelerates age-related bone loss^24^. The activity of mitochondria recovered by 950 nm NIR PBM increases ALP activity, a marker of differentiation, the ultimate goal of stem cells (Fig. 6D). Therefore, 950 nm NIR is highly suitable for postprocessing after thawing cryopreserved stem cells.

The experimental results were collated to identify connections between them and develop a hypothesis about the 950 nm NIR PBM mechanism. The hypothesis is summarized in a schematic diagram explaining the mechanism of PBM in solving post-thawing trauma (Fig. 7). According to our hypothesis, 950 nm NIR PBM activates ETC in mitochondria by activating the CCO. The activation of ETC increases proton transport volume through proton-coupled electron transfer (PCET), increasing MMP^41,42,50^. This increase counters the decrease due to thawing, maintains homeostasis, and prevents apoptosis. In addition, higher MMP increases ATP generation and prevents ATP shortage-induced apoptosis. Moreover, reduction of mitochondrial ROS prevents MMP depolarization caused by mitochondrial ROS, which increase due to thawing. Increased ATP plays a role in gene transcription. Specifically, 950 nm NIR PBM increases cell viability by inhibiting expression of mRNA related to apoptosis. Thus, 950 nm NIR PBM prevents cell death induced by post-thawing trauma in various ways and promotes differentiation of thawed stem cells. In sum, this treatment increases MMP, oxygen consumption of CCO, and mitochondrial mass, hindering mitochondrial dysfunction induced by post-thawing trauma and consequently reducing oxidative stress and cell death. In this study, the limitation is failure to explain the reasons for decrease in ROS. The specific PBM mechanisms for reducing ROS should be revealed in future work.

In conclusion, we discussed cryodamaged stem cells and the mechanism for recovering the damage by PBM. Various indicators have demonstrated that PBM induces normalization of cells damaged by cryopreservation, indicating that PBM can be used as an alternative to antioxidants in post-thawing trauma. PBM is being used as a treatment in various oxidative stress-causing diseases, but the mechanisms of PBM under various cell types and conditions remain unclear. Ultimately, we need an understanding of the more generalized PBM mechanisms associated with different cell types and PBM conditions. Currently, stem cell treatments are a popular research topic, so it is important to treat post-thawing trauma because most methods of preserving stem cells are types of cryopreservation.

## Materials and methods

### Cell culture and cryopreservation

SCAP at a passage of 3–5 were used in this experiment. SCAP were isolated from a human tooth. We obtained Institutional Review Board approval at Seoul National University Hospital (Seoul, South Korea; IRB number CRI05004). Informed consent was obtained from all participants. All experiments were performed in accordance with the Declaration of Helsinki. The manuscript does not contain information or images to identify study participants. Therefore, patient consent for the publication of our experimental data does not seem necessary. Basal media is composed of Alpha-MEM, FBS, and an antibiotic (all of them from Welgene, Korea) at a ratio of 100:10:1. This was used as a complete media. The cells were cultured in an incubator (371 Steri Cycle CO_2_ Incubator, Thermofisher, USA) at a temperature of 37 °C and 5% carbon dioxide. For cryopreservation, EDTA-trypsinized cells were suspended in freezing media of basal media, FBS, and dimethyl sulfoxide (DMSO) (D8418-250ML, Sigma, USA) in a ratio of 6:3:1. A mixture of cells and freezing media was placed in a freezing vial and placed in isopropanol at − 70 °C for 12 h. After 12 h, freezing vials were moved to the liquid nitrogen tank for more than 24 h. Frozen cells were used in experiments after thawing in a 37 °C-waterbath for 2 min.

### PBM system and chemical treatments

The 950 nm NIR device was made with 44 20-mm-spaced LEDs with a wavelength of 950 nm (PV810-3C6W-EDISAA, KAOS, Korea). Well plates were positioned 50 mm above the LEDs. Cells were uniformly irradiated with 950 nm NIR through dispersers from the backlight of an LED TV produced by Samsung Co., Ltd. (Fig. 1A). The 8-bit microcontroller-based (UM_MC95FG308_V3.20_EN, Korea) device operated in CW mode and PW modes with frequencies of 3, 30, 300, and 3000 Hz. The duty cycle was adjusted to 10, 20, 30, 40, and 80% in PW mode. In CW mode, the NIR-irradiated power density was 5.856 (± 0.534) mW/cm^2^ as measured by the power meter (PM-USB-100, Thorlabs, USA). The irradiation flux was 175.68 mJ/cm^2^ in total. When duty cycles were 10, 20, 30, 40, and 80%, the irradiation time was adjusted to 300, 150, 100, 75, and 37.5 s, respectively. In CW mode, the irradiation time was adjusted to 30 s. For experiments, cells contained in a well plate were placed on a transparent acrylic plate in the 37 °C incubator (MyCO_2_, Hanil Science Medical, Korea).

### NAC treatment

The NAC group was created to compare the effects on post-thawing trauma treatment with the NAC of an antioxidant. In NAC groups, the processing conditions for the reagent NAC used to reduce ROS are as follows. NAC (A7250, Sigma, USA) was dissolved in deionized water to 10 mM and was mixed with media to a 0.1 mM final concentration. After thawing, media mixed with NAC was added to cells instead of irradiating. Those cells were incubated at 37 °C for 30 min and washed twice with phosphate buffered saline (PBS). In this state, we incubated cells for 6 h with complete media to measure apoptosis or started other measurements immediately.

### Hydrogen peroxide treatment

The following are conditions under which the cells were treated with hydrogen peroxide to increase ROS. A 3% hydrogen peroxide solution (Sigma) was mixed with media to a solution of 0.25 mM. The cells were seeded on the well plate and incubated for 24 h. After 24 h, the media was suctioned out and replaced with media containing hydrogen peroxide. We cultured this for 1 h at 37 °C and washed it twice with PBS. The complete media was added after washing. After hydrogen peroxide, we irradiated cells with 950 nm NIR or treated cells with NAC.

### Delayed luminescence

For DL measurements, a 24-well plate was set on the acrylic plate (Fig. 1A). A mixture of PBS of 0.5 mL and 1.0 × 10^4^ cells per well was loaded into the well. Here, PBS was used in the culture media due to the fluorescence from FBS. A probe from the power meter was fixed 5 mm above the surface of the mixture. The irradiation followed the procedure above. The intensity of NIR was measured by calculating decay time. Delayed luminescence follows Eq. (1) which is the hyperbolic function^51^. The curves of NIR intensity were fitted in Matlab.

1 $${\text{I}}\left( {\text{t}} \right) \, = {\text{ I}}_{0} / \, \left( {{1 } + {\text{ t}}/\uptau } \right)^{\upbeta } .$$

Here, I_0_ is the initial intensity, β is the delayed luminescence index factor, τ is the delayed luminescence characteristic, and T is the decay time. Decay time was calculated by Eq. (2).

2 $${\text{T }} = \, \left( {{\text{e}}^{{{1}/\upbeta }} - { 1}} \right)\uptau .$$

### Cell viability

The Wst-1 assay kit (EZ-3000, Dogen, Korea) was used to estimate cell viability. After thawing, 1 × 10^4^ cells were seeded in a well of a 96-well plate and treated according to group. The 950 nm group was irradiated by 950 nm NIR, and the NAC-group was treated with 0.1 mM NAC. All wst-1 assays were conducted 24 h after treatments. For the assay, media was removed, and cells were washed with PBS. Wst-1 solution and growth media were mixed at a ratio of 1:10. The working solution was 110 ul per well. The cells were incubated for 1 h in the 37 °C incubator. Formazan dye formed was transported (100 ml each) to a new 96-well plate, and absorbance was measured at 450 nm by ELISA.

### Apoptosis

After post-thawing treatment, 1.0 × 10^5^ cells were seeded in a 24-well plate for each group and incubated for 6 h. After that, cells were harvested and placed in 1.5 ml EP tubes. The harvested cells were washed with PBS. Then, 0.5 ml cold 1× binding buffer and 1.25 µl Annexin V-FITC were added to the cells, according to the manufacturer’s recommendations (EzWay Annexin V-FITC Apoptosis Detection Kit, k29100, Komabiotech, Korea). After being suspended, the sample was incubated for 15 min at room temperature in the dark. After staining, supernatant from centrifugation at 3000 RPM for 5 min was removed from the cells. Then, 0.5 ml cold 1× binding buffer and 10 ul PI were added to the cells. The cells were resuspended in PBS and analyzed immediately via flow cytometry (FACSVerse, Becton Dickinson, USA).

### Intercellular ROS

To measure intercellular ROS, each group of harvested cells was dyed with CM-H2DCFDA (C6827, Invitrogen, USA) immediately after treatment. The cells were suspended in pre-warmed PBS containing dye with a final concentration of 10 μM. The cells were incubated for 15 min at 37 °C, centrifuged for 5 min at 3000 rpm, and the supernatant was removed. The cells were resuspended in culture media and incubated for 20 min at 37 °C. After incubating and centrifuging, the cells with removed supernatant were resuspended in pre-warmed PBS and analyzed via FACS as described above.

### Mitochondrial ROS

MitoSOX (Invitrogen) was used for measuring mitochondrial ROS. According to the protocol, 50 μg of MitoSOX was dissolved in 13 μl of DMSO to prepare a 5 mM MitoSOX stock solution. The stock solution was diluted to a final concentration of 5 μM in Hanks’ balanced salt solution (HBSS). Then, 500 ul of working solution was added to each sample. The cells were incubated for 10 min and washed three times with warm HBSS. After washing, samples were mounted in warm buffer, and their MFIs were immediately measured using FACS.

### Real-time polymerase chain reaction

SCAP cells (5.0 × 10^5^) were seeded in a 4-well plate and harvested 6 h after PBM treatment. The mRNA was extracted (RNeasy Mini kit, Qiagen, Germany), and the cDNA was synthesized using commercial kits (Rever Tra Ace qPCR Master Mix, Toyobo, Japan). mRNA was quantified with a plate reader (Take 3 at Synergy HT, Biotek, USA), reverse transcription was conducted using a thermal cycler (Qiagen, Germany), and real-time PCR was performed using a CFX96 Real-Time System (BioRad, USA). For real-time PCR, Hardshell 96-Well PCR Plates (BioRad, USA), Microseal ‘B’ seals (BioRad, USA), and SYBR green master mix (BioRad, USA) were used. All the processes followed a protocol provided by the manufacturers. The sequence of primers used in quantitative real-time PCR is given in Table 2.

**Table 2:** Sequence of primers used for real-time PCR.

GAPDH forward	5′-CGACCACTTTGTCAAGCTCA-3′
GAPDH reverse	5′-GAGGGTCTCTCTCTTCCTCT-3′
caspase3 forward	5′-GACTCTAGACGGCATCCAGC-3′
caspase3 reverse	5′-CCAGTGAGACTTGGTGCAGT-3′
JNK1 forward	5′-TGTCACACCTGGAAACCTGA-3′
JNK1 reverse	5′-GAAAGGAGAGGGAACGGAAC-3′
RIPK1 forward	5′-CCATGACCCTTTTGCACAGC-3′
RIPK1 reverse	5′-TGGCTGCACTGGAATAAGCA-3′
SOD2 forward	5′-GTTGGCCAAGGGAGATGTTA-3′
SOD2 reverse	5′-TAGGGCTGAGGTTTGTCCAG-3′
SOD3 forward	5′-CCAACAGACACCCTCCACTC-3′
SOD3 reverse	5′-AAGGATGGTGGGTCTCGGTA-3′
PRDX3 forward	5′-GTTGTCGCAGTCTCAGTGGA-3′
PRDX3 reverse	5′-GAGTGCGATGTTCATGTGGC-3′

### Mitochondrial mass

For the mitochondrial mass assay, 1.0 × 10^5^ SCAP cells were used for each sample and were analyzed with Mito-tracker deep red (M22426, Invitrogen, USA). Briefly, a 1 mM stock solution was prepared with 50 μg of Mito-tracker deep red and 92 µl DMSO. The stock solution was diluted with PBS to form a 200 nM working solution. The harvested cells were washed with pre-warmed PBS and centrifuged for 3 min at 3000×*g*. The pre-warmed working solution was added to the cells (with supernatant removed) and incubated for 30 min at 37 °C. After that, the cells were washed with pre-warmed PBS and centrifuged for 3 min at 3000×*g*. MFI was immediately measured with FACS. Then, the cells were resuspended in 20 µl PBS and were put on a confocal dish in 10 µl (101350, SPL, Korea). Images of dyed cells were produced at a rate of 64× using a confocal microscope (LSM800, ZEISS, Germany).

### Mitochondrial membrane potential

MMP was measured using a JC1-mitochondrial membrane potential assay kit (ab113850, Abcam, USA). Harvested cells were washed with PBS. JC-1 diluted with 1× dilution buffer was added to cells to a final concentration of 10 μM. The cells were washed twice with the 1xX dilution buffer after incubating for 30 min. Then, 50 µl of 2.0 × 10^5^ cells was transported to each well of the 96-Well Black Polystyrene Microplate (CLS3603, Corning, USA) followed by 50 µl of the buffer. The relative fluorescence (in RFUs) was measured using a Spark™ 10 M multimode microplate reader. JC1 exists in two forms, aggregate and monomer, depending on the mitochondrial membrane potential, and it emits different fluorescence depending on type. In intact cells, the membrane of the mitochondria is well permeated and exists in the state of JC1-aggregate, producing a 590 nm wavelength fluorescence. In damaged cells, mitochondria remain in the cytoplasm and presents a 529 nm fluorescence in the JC-1 monomer. Thus, the state of the MMP is inferred from the ratio between two intensities of fluorescence, which is called the JC1 ratio and is obtained as JC1 ratio = RFU(Aggregate)/RFU(Monomer)^27^.

### CCO assay

In the CCO activity measurement, there were two groups, the 950 nm group and the control group. Here, 1.0 × 10^6^ cells were used in the measurement. A Triton solution with 1% Triton X-100 (X100, Sigma, USA) was added to the harvested cells and incubated in ice for 10 min. After centrifugation, the cells were diluted in assay buffer and measured with a multimode reader (Spark 10 M, Tecan, Switzerland) at 570 nm. The amount of protein was calculated with a linear regression equation obtained from the standard curve. The slope of the absorbance data in kinetic mode presents a variation of absorbance over time. The slope was divided by the amount of protein, and the result was divided by the molar extinction coefficient provided from Abcam. The final result of the calculation was CCO activity, and the units were units/protein. A definition of “unit” is the amount that oxidizes 1 μmol cytochrome c per minute at 25 °C and pH 7.2.

### ATP assay

The ATP assay employed the ATP assay kit (Abcam, ab83355) to compare the 950 nm group and the control. A standard curve was obtained and used. First, 1.0 × 10^6^ cells were washed with cold PBS. After washing, the 100 µl ATP assay buffer included in the kit was added to lysate cells. After lysis, cells were deproteinized with cold perchloric acid (PCA) and KOH. The hydrogen ion concentration was kept in the range of pH 6.5–8. The ATP working solution was prepared according to the given protocol and added to the cells. They were incubated in a dark room for 30 min at room temperature, and absorbance was measured at 570 nm.

### Alkaline phosphatase (ALP) assay

ALP activity is the main biomarker of osteogenic differentiation. Alkaline Phosphatase Yellow (pNPP) Liquid Substrate (P7998-100ML, Sigma, USA) was used. The ALP assay was implemented on and days 3 and 5 after osteogenic induction. A mixture of the OM ingredient and the complete media in a 1:50 ratio was used for osteogenic induction media. The OM ingredient was prepared with 100 ml deionized water, 10.8 g β-glycerophosphate, 88.1 mg ascorbic acid, and 196.2 μg dexamethasone. Then, 3.0 × 10^3^ cells per well were seeded in a 96-well plate and incubated for 24 h at 37 °C. After that, they were washed with PBS and incubated with osteogenic induction media. At 3 and 5 days after osteogenic induction, ALP activity was measured. The cells were washed twice with a diluted assay buffer (10× Assay Buffer, ThermoFisher) with deionized water in a 1:10 ratio. After washing, 70 µl of the mixture of TritonX-100 (X100, Sigma, USA) and the diluted assay buffer in a ratio of 1:500 was added. The cells were placed in a 4 °C refrigerator for 10 min, and 50 ul diluted TritonX-100 was transferred to a new well plate. Then, 50 µl pNPP solution per well was added to the new well plate with diluted Triton X-100, incubated at 37 °C for 1 h and 15 min, and absorbance was measured by ELISA at 405 nm. Then, 5 µl of the remaining diluted TritonX-100 solution was transferred to a new well plate, and 200 µl of BCA solution (Pierce BCA protein assay reagent A, B, Thermo, USA) was added per well. After incubating at 37 °C for 30 min, the absorbance was measured at 570 nm with ELISA. To determine how much protein was present per well, absorbance data were calibrated using a BCA standard. The blank value was subtracted from the absorbance measured at 405 nm, and the result was divided by the amount of protein.

### Statistical analysis

All experimental data were expressed as mean ± standard deviation (SD). Statistical significance between the experimental groups and control was determined using one-way ANOVA with R-3.6.3 software. Different letters at the tops of columns indicate significant difference at *P* < 0.05 according to Duncan’s multiple range test, performed using R-3.6.3.

### Institutional review board statement

Human stem cells from the apical papilla were isolated from a tooth and we obtained Institutional Review Board approval at Seoul National University Hospital (Seoul, South Korea; IRB number CRI05004).

### Informed consent statement

The manuscript does not contain information or images that could lead to identification of a study participant. Therefore, the consent of patients for publication of our experimental data is not necessary.
